# Prehypertension, Hypertension, and Their Association With Weight‐Adjusted Waist Index in Normoglycemic Japanese Adults: A Cross‐Sectional Study

**DOI:** 10.1155/jdr/9044163

**Published:** 2026-07-23

**Authors:** Jing Fan, Lu Wang, Fawang Li, Shengfei Ruan, Yue Tian, Qing Wang, Zhen Wang

**Affiliations:** ^1^ Department of Laboratory Medicine, Qilu Hospital of Shandong University Dezhou Hospital, Dezhou, Shandong, China; ^2^ Department of Laboratory Medicine, Dezhou Second People′s Hospital, Dezhou, Shandong, China; ^3^ Department of Cardiovascular Medicine, Qilu Hospital of Shandong University Dezhou Hospital, Dezhou, Shandong, China

**Keywords:** hypertension, normoglycemia, obesity, prehypertension, weight-adjusted waist index (WWI)

## Abstract

**Objective:**

Obesity, impaired glucose tolerance, and diabetes significantly increase hypertension risk. Previous studies across various populations have linked the weight‐adjusted waist index (WWI)—a key indicator for assessing obesity—to hypertension. However, this relationship remains unclear specifically in non‐diabetic populations, particularly normoglycemic individuals. Focusing on normoglycemic Japanese adults, the present study explored the correlation of WWI with prehypertension or hypertension.

**Methods:**

Data sourced from the program for medical screening at Murakami Memorial Hospital (Japan) were subjected to secondary analysis, which included 15,464 participants. To quantify the relationship of the WWI with prehypertension (pre‐HTN) or hypertension (HTN), multivariable logistic regression analyses were applied. Restricted cubic spline (RCS) models were further applied to evaluate the dose–response relationship of WWI with the two outcomes. Subgroup analyses were also conducted.

**Results:**

Among 15,453 normoglycemic Japanese adults, the mean WWI increased progressively across blood pressure categories (normal < prehypertension < hypertension). Following adjustment for key confounding variables, WWI was independently correlated with prehypertension (OR = 1.50, 95% CI: 1.39–1.61, *p* < 0.001) and hypertension (OR = 1.74, 95% CI: 1.51–2.00, *p* < 0.001). Curve Fitting confirmed a linear, monotonic positive correlation of WWI with both outcomes (all *p* values for non‐linearity tests > 0.05). Subgroup analyses revealed a stronger association between WWI and prehypertension in females, individuals without fatty liver disease, and current smokers.

**Conclusions:**

Our study demonstrated a positive linear correlation of WWI with both prehypertension and hypertension risk in normoglycemic Japanese individuals.

## 1. Introduction

Globally, hypertension represents the primary modifiable risk factor that can be modified for cardiovascular disease (CVD) and mortality from all causes [1, 2].Hypertension imposes a substantial economic burden, encompassing both direct medical expenditures related to blood pressure management and indirect costs. The latter includes hospitalization expenses due to hypertension‐related complications, along with productivity losses attributable to premature mortality and disability from cardiovascular and renal comorbidities [3].Prehypertension is identified as a condition with a systolic blood pressure (SBP) between 120 and 139 mmHg or a diastolic blood pressure (DBP) between 80 and 89 mmHg, and it represents the preclinical phase preceding hypertension; early recognition and intervention for this condition are therefore essential [4].Consequently, an urgent necessity exists to uncover risk correlates that are both intervenable and measurable in order to reduce the incidence of hypertension. Nevertheless, excess weight gain—particularly when linked to elevated visceral adiposity—constitutes a key contributor to hypertension, with population‐based studies attributing 65%–75% of primary (essential) hypertension risk to this etiology [5].

In recent years, the weight‐adjusted waist index (WWI) has gradually become an important tool for the study of central obesity and its associated health risks [6].Studies have shown that WWI more accurately captures abdominal fat accumulation and shows higher sensitivity in predicting metabolic and cardiovascular risks relative to conventional body mass index (BMI) [7].An increasing number of studies have explored the association between WWI and hypertension [8–10].

However, the correlation of WWI with hypertension in the Japanese population remains to be elucidated. Moreover, it is noteworthy that a proportion of the subjects in these studies had been diagnosed with diabetes. Individuals with diabetes exhibited significantly higher hypertension rates than those without diabetes [11].This observation may be attributed to factors such as insulin resistance and chronic inflammatory state [11, 12].Consequently, the persistent correlation of WWI with hypertension in the nondiabetic population remains uncertain. The objective of the study was to address this knowledge void by examining the correlation of WWI with prehypertension or hypertension in Japanese adults with normoglycemia.

## 2. Methods

### 2.1. Source of the Study Data

Cross‐sectional methodology was utilized for this research, where relevant data were sourced from the publicly accessible DATADRYAD repository (http://www.datadryad.org), specifically accessing the curated dataset published by Okamura et al. (10.5061/dryad.8q0p192) [13], which all researchers can download for free. Initiated as a longitudinal population study at Japan’s Murakami Memorial Hospital (1994–2016), The NAGALA (NAfld in the Gifu Area, Longitudinal Analysis) study has the dual goal of assessing chronic disease determinants and enhancing public health outcomes.

### 2.2. Study Population

To examine how different obesity types affect the risk of developing Type 2 diabetes, Okamura et al. retrospectively analyzed medical records from 20,944 participants who underwent medical examinations at Murakami Memorial Hospital and were registered between 2004 and 2015. Exclusion criteria comprised: (1) alcoholic fatty liver; (2) viral hepatitis (baseline HBsAg or anti‐HCV positivity); (3) taking any medication initially; (4) baseline presence of diabetes mellitus (DM); (5) fasting plasma glucose (FPG) is 6.1 mmol/L or higher; and (6) missing data on covariates. A final dataset of 15,464 participants was obtained. We further excluded 11 participants with missing high‐density lipoprotein cholesterol (HDL‐C) data, yielding a final study cohort of 15,453 subjects. The detailed flow of participant recruitment and exclusion in the original study is available in the corresponding publication.

Ethical approval for the present investigation was provided by the Institutional Review Board of Murakami Memorial Hospital (Approval No. 2018‐09‐01), and written informed consent was obtained from all participants in the original study. Furthermore, the secondary analysis of this data was reviewed and approved by the Ethics Committee of Qilu Hospital of Shandong University Dezhou Hospital (Approval No. 2025153). All analytical procedures strictly complied with the Declaration of Helsinki.

### 2.3. Covariates

The variables included in the database for participants enrolled in the study are as follows: smoking status, triglycerides (TG), DBP, age, waist circumference (WC), weight, alanine aminotransferase (ALT), BMI, *γ*‐glutamyl transpeptidase (GGT), total cholesterol (TC), aspartate transaminase (AST), alcohol consumption, hemoglobin A1c (HbA1c), DM, HDL‐C, sex, SBP, fatty liver, exercise, and FPG. Study participants completed a standardized lifestyle assessment questionnaire. Alcohol intake was assessed based on the mean weekly alcohol consumption during the preceding month. Participants were stratified by (1) alcohol consumption: heavy (> 280 g/week), moderate (141–280 g/week), light (41–140 g/week), or no/minimal (≤ 40 g/week) [14]; (2) smoking status: current, former, or never smokers; and (3) exercise frequency: regular exercisers (> 1 session/week of any modality) [15].A trained technician performed the ultrasound, after which a gastroenterologist reviewed the images and diagnosed fatty liver disease [16].

### 2.4. Measurement of WWI and the Definition of Prehypertension or Hypertension

To find WWI, WC in centimeters was divided by the square root of the weight in kilograms (cm/√kg) [6].Prehypertension and hypertension classifications followed the 2019 Japanese Society of Hypertension (JSH) guidelines [17], defining HTN as systolic BP (SBP) ≥ 140 mmHg and/or diastolic BP (DBP) ≥ 90 mmHg; elevated BP as SBP 130–139 mmHg and/or DBP 80–89 mmHg; and high‐normal BP as SBP 120–129 mmHg with DBP < 80 mmHg. Consistent with previous literature and in accordance with the established criteria of Joint National Committee on Prevention, Detection, Evaluation, and Treatment of High Blood Pressure (JNC 7) of the United States [4, 18], we classify individuals with elevated blood pressure or high‐normal blood pressure as having prehypertension. This condition represents a range of increased cardiovascular risks that necessitate non‐pharmacological interventions. While recognizing evolving classification systems, this combined category aligns with established epidemiological approaches to examine the blood pressure continuum.

### 2.5. Statistical Analysis

Baseline characteristics were summarized as mean ± standard deviation (SD) for normally distributed continuous variables, median (interquartile range [IQR]) for skewed continuous variables, and numbers (proportions) for categorical variables. To evaluate the correlation of baseline WWI with prehypertension/hypertension, we performed univariable and multivariable logistic regression analyses. Covariate selection was guided by causal theory and a directed acyclic graph (DAG; Figure S1) to distinguish confounders from potential mediators. Two models were constructed: an unadjusted model, which assessed the crude association between WWI and prehypertension/hypertension without covariate adjustment, and an adjusted model, which included confounders identified via DAG analysis—namely sex, age, smoking status, aspartate aminotransferase (AST), alcohol consumption, exercise frequency, ALT, fatty liver, and hemoglobin A1c (HbA1c)—to estimate the independent correlation of WWI with the outcomes. Additionally, four‐knot restricted cubic splines (RCS) were employed to assess whether there existed a non‐linear relationship between continuous WWI and the risk of developing hypertension. Exploratory subgroup analyses were performed to investigate potential effect modification of the association between WWI and prehypertension/hypertension, stratified by sex, BMI (≥ 25 versus < 25 kg/m^2^), age (< 65 and ≥ 65 years), smoking status (never, ever, and current), and fatty liver (yes/no). Likelihood ratio tests were used to assess interaction effects, and Benjamini–Hochberg correction (FDR = 0.05) was implemented to account for multiple comparisons, with a corrected *q* value ≤ 0.05 specified as the threshold for a statistically significant result. To verify the robustness of the correlation of WWI with prehypertension/hypertension, a sensitivity analysis was conducted by re‐including the 11 participants with missing HDL‐C data (total sample size = 15,464 after re − inclusion). The same multivariable logistic regression models were reapplied to the full sample (*n* = 15,464) to assess whether the exclusion of participants with missing HDL‐C data had an impact on the primary association results. Statistical analyses were performed using R4.2.2 (http://www.r-project.org/, The R Foundation) and Free Statistics software version 2.2.0. Statistical significance was defined as two‐tailed *p* < 0.05.

## 3. Results

### 3.1. Demographic Features and Baseline Attributes

Baseline characteristics of the 15,453 normoglycemic Japanese participants, stratified according to blood pressure categories, are summarized in Table 1. Specifically, 4412 individuals (28.56%) met the criteria for prehypertension, and 962 (6.22%) were identified as having hypertension. The average age among the included participants was 43.7 ± 8.9 years, with male predominance (54.5%) overall. WWI demonstrated a stepwise increase from normal BP (9.8 ± 0.6 cm/√kg) to prehypertension (10.0 ± 0.6 cm/√kg) and hypertension (10.1 ± 0.5 cm/√kg).

**Table 1:** Baseline characteristics of study population.

Variables	Total (*n* = 15,453)	Normal BP (*n* = 10,079)	Prehypertension (*n* = 4412)	Hypertension (*n* = 962)
Male, *n* (%)	8419 (54.5)	4583 (45.5)	3102 (70.3)	734 (76.3)
Age, (years)	43.7 ± 8.9	42.6 ± 8.6	45.2 ± 9.2	48.2 ± 8.8
Body weight,(kg)	60.6 ± 11.6	57.6 ± 10.1	65.7 ± 11.6	69.5 ± 13.7
WC, (cm)	76.5 ± 9.1	74.0 ± 8.1	80.5 ± 8.7	84.0 ± 9.8
BMI, (kg/m2)	22.1 ± 3.1	21.3 ± 2.6	23.5 ± 3.2	24.8 ± 3.8
Alcohol consumption, *n* (%)				
None	11802 (76.4)	8126 (80.6)	3077 (69.7)	599 (62.3)
Light	1754 (11.4)	1025 (10.2)	596 (13.5)	133 (13.8)
Moderate	1357 (8.8)	695 (6.9)	513 (11.6)	149 (15.5)
Heavy	540 (3.5)	233 (2.3)	226 (5.1)	81 (8.4)
Smoking status, *n* (%)				
Never	9027 (58.4)	6287 (62.4)	2255 (51.1)	485 (50.4)
Past	2949 (19.1)	1589 (15.8)	1106 (25.1)	254 (26.4)
Current	3477 (22.5)	2203 (21.9)	1051 (23.8)	223 (23.2)
Regular exerciser, *n* (%)	2706 (17.5)	1761 (17.5)	773 (17.5)	172 (17.9)
Fatty liver, *n* (%)	2737 (17.7)	1102 (10.9)	1225 (27.8)	410 (42.6)
ALT, (IU/L)	17.0 (13.0, 23.0)	15.0 (12.0, 20.0)	19.0 (14.0, 27.0)	22.0 (16.0, 31.0)
AST,(IU/L)	18.4 ± 8.6	17.4 ± 8.6	20.0 ± 8.4	21.1 ± 8.8
GGT, (IU/L)	15.0 (11.0, 22.0)	14.0 (11.0, 19.0)	19.0 (14.0, 28.0)	22.0 (15.0, 34.0)
HDL‐C, (mg/dL)	56.5 ± 15.6	58.3 ± 15.6	53.7 ± 15.2	51.3 ± 14.4
TC, (mg/dL)	198.2 ± 33.4	194.6 ± 32.7	203.9 ± 33.6	210.1 ± 33.7
TG, (mg/dL)	65.0 (44.0, 99.0)	58.0 (40.0, 86.0)	79.5 (54.0, 120.0)	96.0 (66.0, 144.0)
HbA1c, (%)	5.2 ± 0.3	5.2 ± 0.3	5.2 ± 0.3	5.2 ± 0.3
FPG (mg/dL)	93.0 ± 7.4	91.5 ± 7.2	95.5 ± 7.0	97.1 ± 6.7
WWI,(cm/√kg)	9.9 ± 0.6	9.8 ± 0.6	10.0 ± 0.6	10.1 ± 0.5

*Note:* Data were mean ± SD or median (IQR) for continuous variables or numbers (proportions) for categorical variables.

Abbreviations: ALT, alanine aminotransferase; AST, aspartate aminotransferase; BMI, body mass index; FPG, fasting plasma glucose; GGT, gamma‐glutamyl transferase; HbA1c, hemoglobin A1c; HDL‐C, high‐density lipoprotein cholesterol; Normal BP, Normal blood pressure; TC, total cholesterol; TG, triglyceride; WC, waist circumference; WWI, Weight‐Adjusted Waist Index.

### 3.2. Association Between WWI and Prehypertension or Hypertension

Univariable analysis showed that all factors except exercise were significantly associated with prehypertension and hypertension (all *p* < 0.05; Table S1). Table 2 presents the multivariable‐adjusted correlation of continuous WWI with prehypertension/hypertension. In the unadjusted model, a one‐unit increment in WWI was significantly correlated with elevated odds of prehypertension (OR = 1.74, 95% CI: 1.63–1.85; *p* < 0.001) and hypertension (OR = 2.57, 95% CI: 2.31–2.87; *p* < 0.001). Potential confounders included sex, age, smoking status, AST, alcohol consumption, exercise frequency, ALT, fatty liver, and hemoglobin A1c (HbA1c). These covariates were selected via DAG analysis (see Section 2.5) to identify the minimal sufficient adjustment set, which minimizes confounding bias while avoiding over‐adjustment. Upon adjustment for these relevant confounders, the associations remained statistically significant: each unit increase in WWI was associated with a 50% higher odds of prehypertension (adjusted OR = 1.50, 95% CI: 1.39–1.61; *p* < 0.001) and a 74% higher odds of hypertension (adjusted OR = 1.74, 95% CI: 1.51–2.00; *p* < 0.001).

**Table 2:** Multivariable‐adjusted ORs and 95% CIs of continuous WWI associated with prehypertension and hypertension.

Variable	Unadjusted		Adjusted	
	OR (95% CI)	*p*	OR (95% CI)	*p*
Prehypertension				
WWI	1.74 (1.63~1.85)	< 0.001	1.50(1.39~1.61)	< 0.001
Hypertension				
WWI	2.57 (2.31~2.87)	< 0.001	1.74 (1.51~2.00)	< 0.001

*Note:* The adjusted model adjusts for sex, age, smoking status, AST, alcohol consumption, exercise frequency, ALT, fatty liver, and HbA1c.

Abbreviations: ALT, alanine aminotransferase; AST, aspartate aminotransferase; HbA1c, hemoglobin A1c; WWI, weight‐adjusted waist index.

### 3.3. Non‐Linear Association Between WWI and Prehypertension/Hypertension

**Figure 1:**
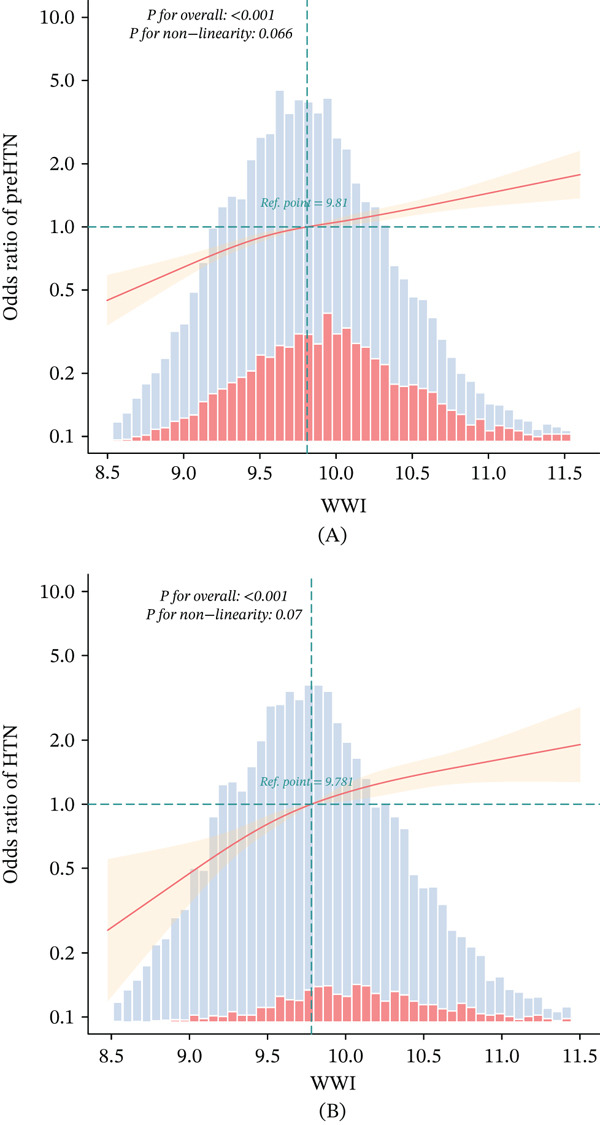
Associations between WWI with prehypertension (A) or hypertension (B). Solid and dashed lines represent the odd ratios and 95% confidence intervals. They were adjusted for sex, age, smoking status, AST, alcohol consumption, exercise frequency, ALT, fatty liver, and HbA1c.

Nonlinear links of continuous WWI with the odds of prehypertension (Figure 1A) and hypertension (Figure 1B) were explored via Curve Fitting, with reference points set at WWI = 9.81 (prehypertension, the median WWI) and WWI = 9.78 (hypertension, the median WWI in the corresponding subgroup), respectively. For both outcomes, the overall association between WWI and disease odds was statistically significant (*p* for overall < 0.001), and no significant non‐linear relationships were observed, with the *p* values for non‐linearity being 0.066 for prehypertension and 0.07 for hypertension. The odds ratios (ORs) for both prehypertension and hypertension showed a consistent upward trend with increasing WWI. These findings indicate that the associations between WWI and prehypertension/hypertension are linear in normoglycemic Japanese adults, with a monotonic increase in the prevalence of prehypertension/hypertension as WWI rises.

### 3.4. Subgroup Analyses

**Figure 2:**
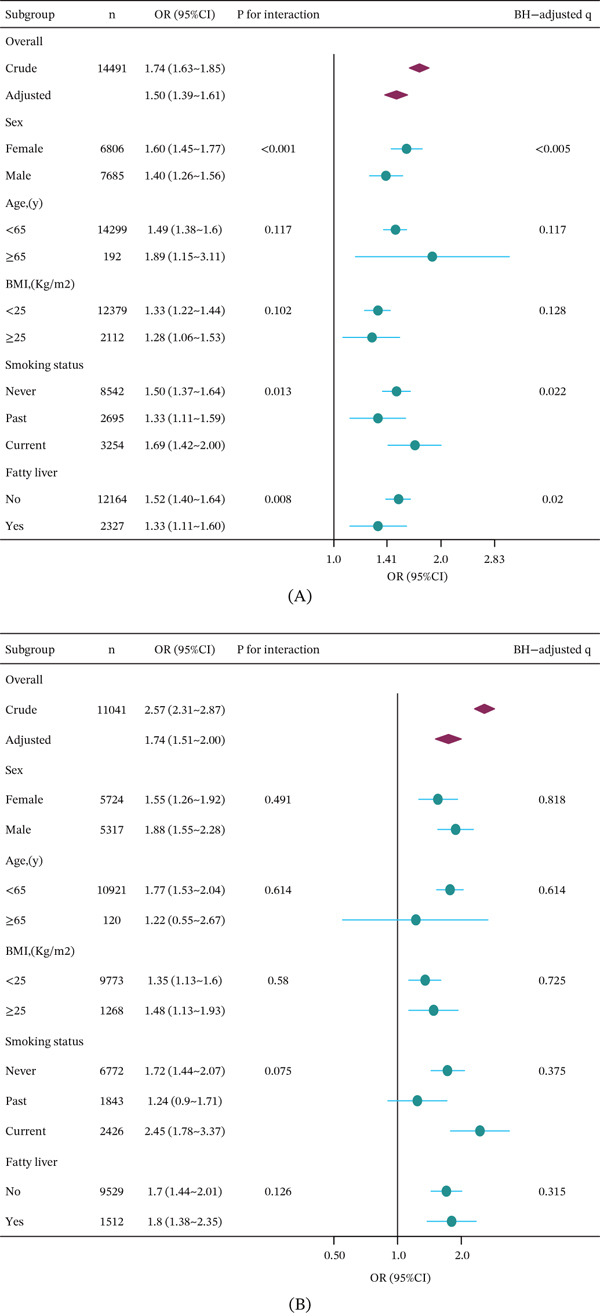
Subgroup analyses of the WWI and prehypertension (A) and hypertension (B).

For the pre‐hypertension outcomes (Figure 2A): After FDR correction, interactions remained statistically significant in the subgroups of gender (original *p* < 0.001, corrected *q* < 0.005), smoking status (original *p* = 0.013, corrected *q* = 0.022), and fatty liver (original *p* = 0.008, corrected *q* = 0.02). Stratified analysis revealed that females (OR = 1.60, 95% CI: 1.45–1.77), current smokers (OR = 1.69, 95% CI: 1.42–2.00), and individuals without fatty liver (OR = 1.52, 95% CI: 1.40–1.64) exhibited stronger associations between WWI and prehypertension compared with corresponding subgroups (males OR = 1.40, 95% CI: 1.26–1.56; OR = 1.50, 95% CI: 1.37–1.64 for never smokers; OR = 1.33, 95% CI: 1.11–1.60 for those with fatty liver). Statistical significance was not attained for the interaction terms of age (unadjusted *p* = 0.117, adjusted *q* = 0.117) and BMI (unadjusted *p* = 0.102, adjusted *q* = 0.128) after adjustment for confounding factors.

For the hypertension outcome (Figure 2B): The unadjusted *p* values for interactions across all subgroups were > 0.05. After the FDR adjustment, the adjusted *q* values for each subgroup remained > 0.05, indicating no significant effect modification.

### 3.5. Sensitivity Analysis

Re‐including the 11 participants with missing HDL‐C data did not alter the primary association results (Table S2). The adjusted ORs for WWI with prehypertension and hypertension were 1.50 and 1.74, respectively—exactly consistent with the original findings (1.50 and 1.74) and accompanied by overlapping 95% confidence intervals and *p* < 0.001 for all analyses.

## 4. Discussion

In this study of 15,453 Japanese adults with normal blood glucose levels, we observed a progressive increase in the mean WWI with increasing blood pressure categories. After adjusting for potential confounders, WWI was positively correlated with both prehypertension and hypertension. This association exhibited a linear, monotonically increasing trend across the entire range of WWI values. The association between WWI and prehypertension was stronger in female participants, those without fatty liver disease, and current smokers (statistically significant after adjusting for BH interaction), whereas the association with hypertension remained consistent across all examined subgroups.

We carried out a sensitivity analysis via re‐inclusion of participants with missing HDL‐C data, and the congruent outcomes of multiple logistic regression confirmed that our results were robust. Given the negligible count of re‐enrolled subjects, we focused on validating the key association, which constitutes a more efficient strategy to assess the stability of findings.

The extant studies on the correlation of WWI with the risk of hypertension have reported analogous results to the present study. A study of US adults revealed a positive correlation of WWI with the prevalence of hypertension, particularly among male subjects under the age of 60 [19].A subsequent study concentrating on US adults over the age of 60 yielded analogous results [9].In a Chinese population, Liu et al. assessed the association of six alternative IR indices with hypertension risk and confirmed that WWI was independently associated with an increased risk of developing hypertension [20].Findings from a cohort study in rural China indicated the presence of a non‐linear correlation of WWI with HTN [8].A cross‐sectional and cohort study from the China Health and Retirement Longitudinal Study revealed a positive association of WWI with SBP and DBP. In longitudinal cohort studies, WWI demonstrated a linear correlation with new‐onset hypertension, exhibiting a higher predictive value compared with WC and BMI [10].In this study of normoglycemic Japanese adults, the observed positive WWI–hypertension association adds to the growing body of evidence from American and Chinese populations, supporting the consistency of the link between WWI and blood pressure abnormalities across distinct East Asian and Western populations examined to date. Future prospective studies with multi‐ethnic cohorts are needed to further verify the robustness of this association and explore the potential utility of WWI as an adiposity‐related indicator in broader populations.

Although DM and impaired glucose tolerance significantly increase the risk of hypertension [11], there is a relative lack of studies in the normoglycemic population, and they are susceptible to the confounding effects of medications (e.g., antihypertensive and hypoglycemic agents). In the present study, we excluded those taking medication at baseline and demonstrated an independent positive association of WWI with both pre‐HTN and HTN in a Japanese normoglycemic population. This suggests that abdominal obesity‐related mechanisms (e.g., visceral adiposity‐induced chronic low‐grade inflammation, impaired renal‐pressure natriuresis, sympathetic nervous system activation, or endothelial dysfunction) may contribute to hypertension development independent of dysglycemia [5, 21–24].

Based on the JSH 2019 standard and considering the research evidence that blood pressure within the range of 120–139/80–89 mmHg considerably elevates the likelihood of developing hypertension and CVD [25–27], the current investigation categorized prehypertension as falling within this blood pressure range. Cuspidi et al. [28] emphasized the importance of early identification of this stage. Analyses of RCS revealed a gradual increase in the prevalence of prehypertension with rising WWI. This also offers an important insight: particularly amid growing emphasis on health management, closer blood pressure monitoring could be considered for individuals with elevated WWI—even when their blood glucose levels are within the normal range. Of note, this proposition requires further validation in diverse populations and prospective studies.

This study conducted exploratory subgroup analyses based on cross‐sectional data from Japanese individuals with normal blood glucose levels to identify potential effect modifiers linking WWI to prehypertension and hypertension. After FDR correction, significant subgroup heterogeneity was observed only in the prehypertension outcome, while no similar pattern was noted in the hypertension outcome. This finding provides a hypothesis direction for subsequent targeted research.

In the gender subgroup analysis, the association strength between WWI and prehypertension was stronger in women than in men, consistent with the gender‐specific characteristics of body fat distribution in East Asian populations. Previous studies indicate that East Asian women are more prone to abdominal fat accumulation [29], and excessive abdominal fat (particularly visceral fat) can disrupt blood pressure regulation mechanisms through pathways such as inducing insulin resistance, activating the sympathetic nervous system, and promoting the release of inflammatory factors, thereby increasing the risk of prehypertension [5].In contrast, men exhibit relatively dispersed body fat distribution, with abdominal fat exerting a comparatively milder impact on metabolism and blood pressure. Within the smoking status subgroup, current smokers exhibited a stronger association between WWI and prehypertension. Smoking directly damages vascular endothelial function, reducing vasodilation capacity [30].Meanwhile, the abdominal fat accumulation corresponding to elevated WWI exacerbates oxidative stress and inflammatory responses. The joint influence of these two factors might further compromise the body’s blood pressure homeostasis. In the fatty liver subgroup, the correlation of WWI with prehypertension was more pronounced in individuals without fatty liver disease, potentially reflecting an “effect saturation” phenomenon. Fatty liver itself is a key marker of metabolic dysfunction, often accompanied by multiple abnormalities such as insulin resistance and dyslipidemia. These abnormalities have become dominant factors influencing blood pressure, masking the additional association strength of WWI on blood pressure. In contrast, individuals without fatty liver and with normal blood glucose levels exhibit relatively stable metabolic states. As a surrogate marker for visceral fat, WWI shows a more pronounced association strength with prehypertension in this group.

Notably, no significant heterogeneity in hypertension outcomes was observed across any subgroup, potentially reflecting the chronic nature of hypertension. As a long‐term condition, hypertension develops through the cumulative effects of multiple factors over time. The impact of WWI may be diluted by other persistent risk factors (e.g., chronic high‐salt diet, genetic predisposition), resulting in less pronounced differences in association strength across subgroups.

## 5. Limitations

Several limitations should also be acknowledged in the present study. (1) Study Design Limitations: This cross‐sectional study can only reveal the association of WWI with prehypertension/hypertension without establishing causality or directionality. Consequently, any screening and prevention recommendations based on these findings require validation through prospective cohort studies. (2) Subgroup Analysis Attribute Limitations: Subgroup analyses in this study were exploratory and not pre‐specified, introducing potential data‐driven bias. Despite multiple testing corrections using the Benjamini–Hochberg method, false positive results remain possible. All subgroup heterogeneity findings are hypothesis‐generating and require independent validation. (3) Insufficient Sample Size in Some Subgroups: The subgroup aged ≥ 65 years had a small sample size (hypertension group: *n* = 120; prehypertension group: *n* = 192), resulting in low statistical power for this subgroup. The 95% confidence intervals for interaction estimates were wide, limiting the stability and reliability of the results. Definitive conclusions cannot be drawn, and validation in a larger elderly population is required. (4) Unmeasured Confounders and Missing Mediators: While common confounders (e.g., age, metabolic parameters) were adjusted for, unmeasured factors—including dietary patterns, genetic background, and psychological factors—may still confound the WWI–blood pressure association and subgroup results. Visceral obesity (as reflected by BMI) induces hypertension through pathways involving chronic inflammation and insulin resistance; however, the absence of these unmeasured mediating variables in the dataset hinders our ability to elucidate the biological mechanisms underlying the association between WWI and blood pressure. (5) Selection Bias Limitations: The voluntary NAGALA cohort overrepresents health‐conscious individuals, whose lifestyle habits may skew the observed WWI–blood pressure association and limit applicability to the general Japanese population. (6) Population Limitations: Restricted to Japanese normoglycemic individuals, these findings cannot be extrapolated to other ethnicities or those with abnormal glucose levels, requiring multi‐center, multi‐ethnic validation.

Notwithstanding these limitations, our findings offer valuable implications for both research and clinical practice. On one hand, they highlight the need to develop more standardized protocols for the measurement and interpretation of WWI in clinical settings. On the other hand, future studies should place greater emphasis on blood pressure monitoring in a frequently overlooked population—normoglycemic individuals with elevated WWI.

## 6. Conclusion

Among Japanese individuals with normal blood glucose levels, the prevalence of prehypertension and hypertension increases in parallel with rising weight‐adjusted waist circumference index. Future studies should prospectively assess whether WWI‐guided interventions reduce hypertension incidence, examine longitudinal relationships between WWI reduction and blood pressure changes, and identify effect modifiers in the WWI–hypertension association.

## Author Contributions

J.F.: conceptualization, writing – original draft. L.W.: data curation, formal analysis. F.L.: investigation, visualization. S.R.: conceptualization. Y.T.: resources, project administration. Q.W.: methodology, critical revision, supervision, correspondence. Z.W.: supervision, writing – review and editing, correspondence. J.F. and L.W. have contributed equally to this work.

## Funding

No funding was received for this manuscript.

## Disclosure

All authors approved the final manuscript.

## Ethics Statement

The ethical committee at Murakami Memorial Hospital reviewed and approved the original study involving human participants (ID: 2018‐09‐01). Written informed consent was obtained from the participants for their involvement in that study. The present secondary analysis of the publicly available Dryad dataset received separate ethical approval from the Ethics Committee of Qilu Hospital of Shandong University Dezhou Hospital (Approval No. 2025153). All procedures were conducted in accordance with the Declaration of Helsinki.

## Conflicts of Interest

The authors declare no conflicts of interest.

## Supporting Information

Additional supporting information can be found online in the Supporting Information section.

## Supporting information


**Supporting Information 1** Figure S1: Directed acyclic graph.

**Supporting Information 2** Table S1: Results of univariable analysis of prehypertension and hypertension.

**Supporting Information 3** Table S2: Multivariate logistic regression after re‐including 11 excluded subjects.

## Data Availability

The datasets presented in this study are available online repositories. The names of the repository and accession numbers can be found in the article.
