# Supplementary material for: Prehypertension, Hypertension, and Their Association With Weight‐Adjusted Waist Index in Normoglycemic Japanese Adults: A Cross‐Sectional Study
Source: J Diabetes Res. 2026 Jul 23;2026:9044163. doi: 10.1155/jdr/9044163 (PMC13396699; doi:10.1155/jdr/9044163)
Supplement: Supplementary file 1 — Supporting Information 1 Figure S1: Directed acyclic graph. [file JDR-2026-9044163-s002.docx]

**Supplementary Figure 1.  Directed Acyclic Graph**


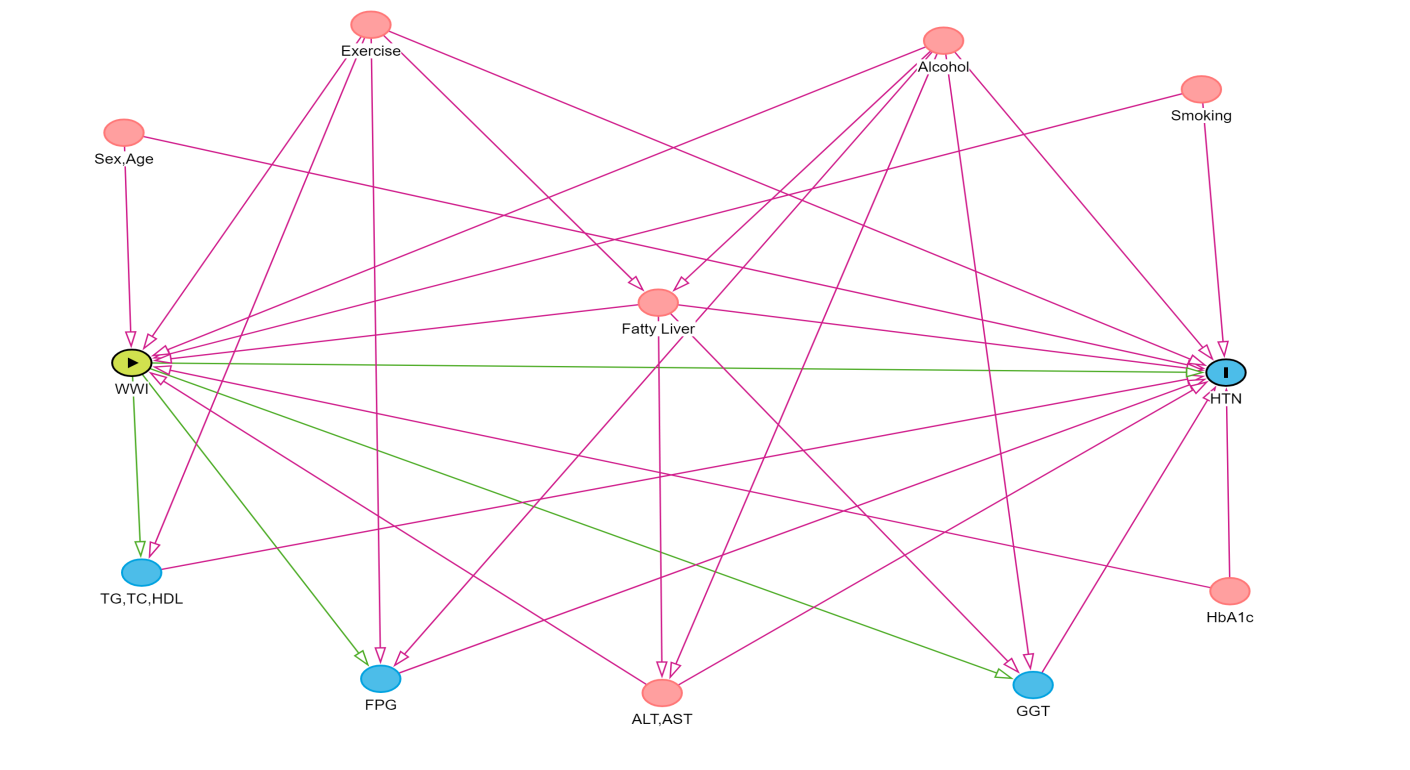


This directed acyclic graph illustrates the hypothesized associations among covariates, the primary exposure (weight-adjusted waist index, WWI; yellow circle), and the outcome (prehypertension/hypertension [HTN]; dark blue circle): pink circles represent confounders (ancestors of both exposure and outcome, including sex/age, exercise, alcohol, smoking, fatty liver, and ALT/AST), while blue circles denote mediators (causal determinants of the outcome along the exposure-outcome pathway, including TG/TC/HDL, FPG, and GGT). Green lines indicate hypothesized causal paths (from exposure to mediators, and mediators to outcome), and pink lines represent potential biasing paths (from confounders to exposure, confounders to outcome, or confounders to mediators). The minimally sufficient adjustment set—determined using DAG principles to minimize confounding bias when estimating the WWI-HTN association—comprises the confounders (pink circles); mediators (blue circles) were excluded from adjustment to avoid overadjustment bias in the exposure-outcome association.
