# Supplementary material for: Prehypertension, Hypertension, and Their Association With Weight‐Adjusted Waist Index in Normoglycemic Japanese Adults: A Cross‐Sectional Study
Source: J Diabetes Res. 2026 Jul 23;2026:9044163. doi: 10.1155/jdr/9044163 (PMC13396699; doi:10.1155/jdr/9044163)
Supplement: Supplementary file 2 — Supporting Information 2 Table S1: Results of univariable analysis of prehypertension and hypertension. [file JDR-2026-9044163-s003.docx]

**Supplementary Table 1: Results of univariable analysis of prehypertension and hypertension.**

| Variable | Prehypertension | | Hypertension | |
| --- | --- | --- | --- | --- |
|  | OR (95%CI) | p value | OR (95%CI) | p value |
| Sex, n (%) | 2.84 (2.63~3.06) | <0.001 | 3.86 (3.31~4.5) | <0.001 |
| Age, (years) | 1.03 (1.03~1.04) | <0.001 | 1.07 (1.06~1.08) | <0.001 |
| BMI, (kg/m2) | 1.3 (1.28~1.32) | <0.001 | 1.44 (1.41~1.47) | <0.001 |
| WC, (cm) | 1.1 (1.09~1.1) | <0.001 | 1.14 (1.13~1.15) | <0.001 |
| Body Weight | 1.07 (1.07~1.07) | <0.001 | 1.09 (1.08~1.1) | <0.001 |
| Alcohol consumption |  |  |  |  |
| None | ref |  | ref |  |
| Light | 1.54 (1.38~1.71) | <0.001 | 1.76 (1.44~2.15) | <0.001 |
| Moderate | 1.95 (1.73~2.2) | <0.001 | 2.91 (2.39~3.54) | <0.001 |
| Heavy | 2.56 (2.12~3.09) | <0.001 | 4.72 (3.61~6.15) | <0.001 |
| Smoking status,n (%) |  |  |  |  |
| Never | ref |  | ref |  |
| Past | 1.94 (1.77~2.12) | <0.001 | 2.07 (1.76~2.44) | <0.001 |
| Current | 1.33 (1.22~1.45) | <0.001 | 1.31 (1.11~1.55) | 0.001 |
| Regular exerciser,n (%) |  |  |  |  |
| No | ref |  | ref |  |
| Yes | 1 (0.91~1.1) | 0.944 | 1.03 (0.87~1.22) | 0.751 |
| Fatty liver,n (%) |  |  |  |  |
| No | ref |  | ref |  |
| Yes | 3.13 (2.86~3.43) | <0.001 | 6.05 (5.25~6.98) | <0.001 |
| ALT, (IU/L) | 1.04 (1.03~1.04) | <0.001 | 1.04 (1.04~1.04) | <0.001 |
| AST,(IU/L) | 1.05 (1.05~1.06) | <0.001 | 1.05 (1.04~1.06) | <0.001 |
| GGT, (IU/L) | 1.03 (1.03~1.03) | <0.001 | 1.03 (1.03~1.03) | <0.001 |
| HDL-C, (mg/dL) | 0.98 (0.98~0.98) | <0.001 | 0.97 (0.96~0.97) | <0.001 |
| TC, (mg/dL) | 1.01 (1.01~1.01) | <0.001 | 1.01 (1.01~1.02) | <0.001 |
| TG, (mg/dL) | 1.01 (1.01~1.01) | <0.001 | 1.01 (1.01~1.01) | <0.001 |
| HbA1c, (%) | 1.71 (1.53~1.92) | <0.001 | 2.25 (1.83~2.77) | <0.001 |
| FPG ,(mg/dL) | 1.08 (1.08~1.09) | <0.001 | 1.12 (1.11~1.13) | <0.001 |
| WWI,(cm/√kg) | 1.74 (1.63~1.85) | <0.001 | 2.57 (2.31~2.87) | <0.001 |

BMI, body mass index; WC, waist circumference;ALT, alanine aminotransferase; AST, aspartate aminotransferase;GGT, gamma glutamyl transferase; HDL-C, high‐density lipoprotein cholesterol; TC, total cholesterol; TG, triglyceride; HbA1c, hemoglobin A1c; FPG, fasting plasma glucose; WWI, Weight-Adjusted Waist Index.
