# Supplementary material for: Prehypertension, Hypertension, and Their Association With Weight‐Adjusted Waist Index in Normoglycemic Japanese Adults: A Cross‐Sectional Study
Source: J Diabetes Res. 2026 Jul 23;2026:9044163. doi: 10.1155/jdr/9044163 (PMC13396699; doi:10.1155/jdr/9044163)
Supplement: Supplementary file 3 — Supporting Information 3 Table S2: Multivariate logistic regression after re‐including 11 excluded subjects. [file JDR-2026-9044163-s001.docx]

**Supplementary Table 2:Multivariate logistic regression after re-including 11 excluded subjects**

| Variable | Unadjusted | | | Adjusted | | |
| --- | --- | --- | --- | --- | --- | --- |
|  | OR (95%CI) | | *P* | OR (95%CI) | *P* | |
| Pre-HTN |  | |  |  |  | |
| WWI | 1.74 (1.63~1.84) | <0.001 | | 1.50(1.39~1.61) | | <0.001 |
| HTN |  | |  |  |  | |
| WWI | 2.57 (2.31~2.87) | | <0.001 | 1.74 (1.51~2.00) | <0.001 | |

The adjusted model adjusts for age, sex, smoking status, alcohol consumption, regular exercise, ALT, AST, HbA1c, and fatty liver.

WWI, weight-adjusted waist index; ALT, alanine aminotransferase; AST, aspartate aminotransferase; HbA1c, hemoglobin A1c.
